# Association of PI-RADS Score with Clinically Significant Prostate Cancer and Histopathological Upgrading in Patients Undergoing Radical Prostatectomy: A Retrospective Single-Centre Study

**DOI:** 10.3390/jcm15176730

**Published:** 2026-08-30

**Authors:** Hubert Andrzej Krzepkowski, Tomasz Ząbkowski, Andrzej Wieczorek, Maciej Walędziak, Tomasz Waldemar Kamiński, Hubert Dąbrowski, Tomasz Syryło

**Affiliations:** 1 Department of General, Oncological and Functional Urology, Military Institute of Medicine-National Research Institute, 04-141 Warsaw, Poland; 2 Department of General, Oncological, Metabolic and Thoracic Surgery, Military Institute of Medicine-National Research Institute, Szaserów 128 St., 04-141 Warsaw, Poland; 3 Faculty of Medicine, University of Warsaw, 04-141 Warsaw, Poland; 4 VERSITI Blood Research Institute, 8727 W Watertown Plank Road, Wauwatosa, WI 53226, USA; 5 Psychiatric Ward of the Mental Health Center in Kielce, 59 Kosucińskiego, 25-450 Kielce, Poland; 6 Świętokrzystkie Center of Psychiatry in Morawica, 5 Spacerowa, 26-026 Morawica, Poland

**Keywords:** prostate cancer, multiparametric MRI, PI-RADS, Gleason upgrading, radical prostatectomy, MRI-targeted biopsy, risk stratification

## Abstract

**Background and Objectives**: Multiparametric magnetic resonance imaging (mpMRI) is widely used in prostate cancer diagnosis, but biopsy findings may still underestimate tumor aggressiveness. This study evaluated the association of Prostate Imaging Reporting and Data System (PI-RADS) assessment with clinically significant prostate cancer (csPCa) and histopathological upgrading after radical prostatectomy. **Methods**: The retrospective analysis comprised 564 patients who underwent mpMRI–transrectal ultrasound fusion-guided biopsy followed by radical prostatectomy between 2021 and 2025. All included patients subsequently underwent radical prostatectomy, so the cohort represents a surgically selected population. Clinical variables, PI-RADS score, biopsy ISUP Grade Group, and final prostatectomy pathology were assessed. Clinically significant disease was defined as ISUP Grade Group ≥ 2. Histopathological upgrading was defined as an increase of at least one ISUP Grade Group between biopsy and final prostatectomy pathology. **Results**: csPCa was found in 385 patients (68.3%). Patients with csPCa had higher prostate-specific antigen density (PSAD) values than those without csPCa. PI-RADS score was significantly associated with csPCa and remained independently associated with csPCa in multivariable analysis. Histopathological upgrading occurred in 164 patients (29.1%), and PI-RADS score was also independently associated with upgrading. Model discrimination was limited. **Conclusions**: Preoperative mpMRI with PI-RADS assessment was associated with both csPCa and biopsy underestimation. Although PI-RADS was independently associated with both outcomes, its modest discriminatory performance indicates that it should be interpreted together with other clinical and pathological parameters rather than as a standalone predictive tool.

## 1. Introduction

Prostate cancer is one of the most frequently diagnosed cancers in men worldwide and remains an important cause of cancer-related morbidity and mortality. Recent epidemiological data indicate a steady increase in incidence, partly driven by population aging and improved detection, whereas the disease continues to be a leading cause of cancer death among men, underscoring the ongoing need for effective strategies for prevention, early diagnosis, and treatment [1]. Although widespread PSA testing has improved prostate cancer detection, it has also increased the detection of clinically insignificant disease and raised concerns about overdiagnosis and subsequent overtreatment [2,3]. Recently, multiparametric magnetic resonance imaging (mpMRI) of the prostate has emerged as a key tool for the diagnosis of prostate cancer. mpMRI combines anatomical and functional imaging techniques, including T2-weighted, diffusion-weighted, and dynamic contrast-enhanced imaging, thereby improving lesion detection, localization, and risk stratification [4,5]. The introduction of the Prostate Imaging Reporting and Data System (PI-RADS) further standardized MRI interpretation and reporting, improving consistency and clinical applicability [6]. Accumulating evidence supports the use of mpMRI for identifying clinically significant prostate cancer. Compared with systematic biopsy alone, mpMRI demonstrates higher sensitivity for identifying clinically relevant lesions and enables more accurate targeting during biopsy. Importantly, its high negative predictive value permits the safe exclusion of significant disease in a subset of patients, thereby reducing unnecessary invasive procedures. Consequently, mpMRI has become an important component of the modern diagnostic pathway for prostate cancer [7]. Its diagnostic performance is particularly relevant for the detection of clinically significant disease and may help reduce unnecessary biopsies [8]. Therefore, mpMRI is recommended as an important component of the diagnostic pathway for men with suspected prostate cancer [9]. Despite these advances, some important limitations remain. A major challenge in prostate cancer diagnosis is biopsy underestimation, defined as discordance between biopsy findings and the final pathological assessment following radical prostatectomy. Underestimation may lead to inappropriate risk stratification and suboptimal treatment decisions [10]. Even with MRI-targeted biopsy approaches, a proportion of patients initially classified as having low- or intermediate-risk disease are subsequently found to harbor more aggressive cancers on final histopathology [11]. Although mpMRI has demonstrated significant potential for risk assessment, its ability to reliably predict biopsy underestimation remains unclear. Furthermore, variability in MRI interpretation, lesion characteristics, and patient-related factors may influence diagnostic accuracy and clinical outcomes [12].

Further research is needed to clarify the relationship between prostate MRI findings, clinically significant prostate cancer, and histopathological upgrading between biopsy and radical prostatectomy. This study aimed to evaluate the association of PI-RADS score and selected clinical parameters with clinically significant prostate cancer and histopathological upgrading between biopsy and final radical prostatectomy specimens in patients undergoing radical prostatectomy after MRI–TRUS fusion-guided biopsy.

## 2. Materials and Methods

This retrospective cohort study included 564 patients who underwent multiparametric magnetic resonance imaging–transrectal ultrasound (mpMRI–TRUS) and transperineal mpMRI–TRUS fusion-guided targeted biopsy with additional perilesional sampling around the MRI-visible lesions, followed by radical prostatectomy. Biopsies were performed predominantly using a BK 5000 ultrasound system (GE HealthCare, Chicago, IL, USA) at our institution, while the specific fusion platforms used at external centers were not consistently documented. An average of 4–6 targeted cores were obtained from each MRI-visible lesion, with additional perilesional cores obtained around the target and submitted separately for histopathological assessment. Systematic biopsy cores were not routinely obtained. The biopsy protocol remained unchanged throughout the study period from 2021 to 2025. Operator experience in performing the biopsies was not systematically recorded in the retrospective dataset. All procedures were performed at a tertiary referral center from 2021 through 2025. Patients were included only if radical prostatectomy was performed after biopsy, as final whole-gland prostatectomy histopathology was required as the reference standard for assessment of biopsy upgrading. Patients were included consecutively according to the study eligibility criteria. Cases with insufficient documentation of preoperative clinical or imaging parameters required for the present analysis were excluded. In particular, cases with incomplete mpMRI documentation were excluded. The final study cohort comprised 564 patients. The analysis included patient age, serum PSA concentration, prostate volume, and digital rectal examination (DRE) findings. PSAD was derived by dividing serum PSA by prostate volume and was expressed in ng/mL/cc.

Radiological assessment was based on mpMRI, and lesions were classified according to the PI-RADS v2.1, with scores ranging from 1 to 5. MRI examinations were interpreted by multiple radiologists, with each examination assessed by a single radiologist. MRI examinations were performed at multiple centers using different MRI systems. Detailed information regarding magnetic field strength (1.5 T versus 3 T) was not consistently available in the retrospective dataset. MRI readers had access to PSA and DRE findings but did not have access to biopsy results, as MRI assessment preceded biopsy. MRI examinations were interpreted as part of routine clinical practice. The radiologists were predominantly from our institution and generally had several years of experience in prostate MRI interpretation. No central rereading was performed for the present study, and interobserver agreement was not assessed. Histopathological evaluation included ISUP Grade Group assessment of both biopsy specimens and radical prostatectomy samples. Histopathological assessment was performed by specialist pathologists. Radical prostatectomy specimens were evaluated using whole-mount sections. No central pathological rereview was performed for the present study. Pathological assessment was performed with access to the available clinical information, including MRI reports, PSA, DRE findings, and the operative report. Gleason scores were classified and stratified according to the International Society of Urological Pathology (ISUP) Grade Group system. csPCa was defined as ISUP Grade Group ≥ 2 (Gleason score ≥ 3 + 4), whereas ISUP Grade Group 1 (Gleason score 3 + 3) was considered clinically insignificant disease. Concordance between biopsy and prostatectomy specimens was assessed, and cases were classified as concordant, upgraded, or downgraded based on ISUP Grade Group changes. Histopathological upgrading was defined as an increase of at least one ISUP Grade Group from biopsy to final radical prostatectomy pathology. Downgrading was defined as a decrease of at least one ISUP Grade Group. Cases with no change in ISUP Grade Group were classified as concordant. The primary objective of this study was to assess the association of PI-RADS score and selected clinical parameters with csPCa and histopathological upgrading between biopsy and final radical prostatectomy pathology.

Statistical analyses were performed using GraphPad Prism 11.0 (GraphPad Software, La Jolla, CA, USA), when bootstrap validation and additional regression diagnostics were performed in Python 3.11 (Python Software Foundation, Wilmington, DE, USA). The distribution of continuous variables was assessed, and data were presented as the mean ± standard deviation (SD) or the median with interquartile range (IQR), as appropriate. Categorical variables are presented as absolute numbers and percentages.

Between-group comparisons were performed using an unpaired Student *t* test for normally distributed continuous variables or the Mann–Whitney U test for non-normally distributed data. Categorical variables are compared with the chi-square test or Fisher’s exact test, as appropriate.

csPCa was defined as an ISUP Grade Group ≥ 2 (Gleason score ≥ 3 + 4). Histopathological upgrading was defined as an increase of at least one ISUP Grade Group from biopsy to final radical prostatectomy pathology.

Univariable logistic regression analyses were performed to evaluate associations with biopsy-detected csPCa and with histopathological upgrading on final radical prostatectomy pathology. Variables included age, serum PSA level, prostate volume, PSAD, PI-RADS score, and baseline biopsy Gleason score. Baseline biopsy Gleason score was included as a categorical variable in the upgrading model. Because the retrospective dataset did not consistently distinguish Gleason 3 + 4 from 4 + 3 disease, baseline biopsy Gleason score rather than the complete ISUP Grade Group classification was used in this analysis. Multivariable logistic regression models were constructed separately for csPCa and histopathological upgrading. For the upgrading analysis, baseline biopsy Gleason score was additionally included as a categorical covariate. To assess the incremental contribution of PI-RADS, nested models without and with PI-RADS were compared. PI-RADS was analyzed as an ordinal variable, with odds ratios expressed per 1-point increase. Model discrimination was assessed using ROC analysis and AUC. The odds ratios (ORs) with 95% confidence intervals (CIs) were calculated. Multicollinearity among variables included in the multivariable models was assessed using variance inflation factors (VIFs). A VIF < 5 was considered to indicate the absence of substantial multicollinearity. Age and PI-RADS data were complete, whereas missing values were present for PSA and prostate volume. Internal validation of the multivariable models was performed using 1000 bootstrap resamples to estimate optimism-corrected AUC values. No imputation was performed, and regression analyses were based on complete cases.

The discriminatory performance of the multivariable models was assessed using receiver operating characteristic (ROC) curve analysis, and the area under the curve (AUC) was calculated as a measure of model discrimination. DRE findings were not included in the multivariable analysis because of incomplete standardization. Given the retrospective, single-center design, model performance should be interpreted cautiously. All statistical tests were two-sided, and *p* < 0.05 was considered statistically significant. The study was conducted in accordance with the Declaration of Helsinki. The Bioethics Committee of the Military Institute of Medicine-National Research Institute in Warsaw reviewed the study protocol and determined that the study did not require ethical approval (decision No. KB/30/26, 20 May 2026). This determination was obtained prior to the commencement of the present retrospective analysis.

## 3. Results

The study cohort comprised 564 patients who underwent MRI-TRUS fusion biopsy followed by radical prostatectomy. Clinically significant prostate cancer (csPCa; ISUP ≥ 2) was identified in 385 patients (68.3%), whereas 179 patients (31.7%) had Gleason score ≤ 3 + 3 disease. Most patients had PI-RADS 4 or 5 lesions on preoperative mpMRI. Detailed baseline characteristics of the cohort, including age, PSA level, prostate volume, PSAD, ISUP Grade Group, and PI-RADS distribution, are presented in Table 1.

Compared with patients without csPCa, those with csPCa exhibited significantly higher PSAD values (0.246 vs. 0.204 ng/mL/cc; *p* < 0.01). Prostate volumes also tended to be lower in the csPCa-positive group, although the difference was not statistically significant. No significant differences were observed in age or PSA levels between groups. A detailed comparison of csPCa-negative and csPCa-positive patients is shown in Table 2.

**Figure 1:**
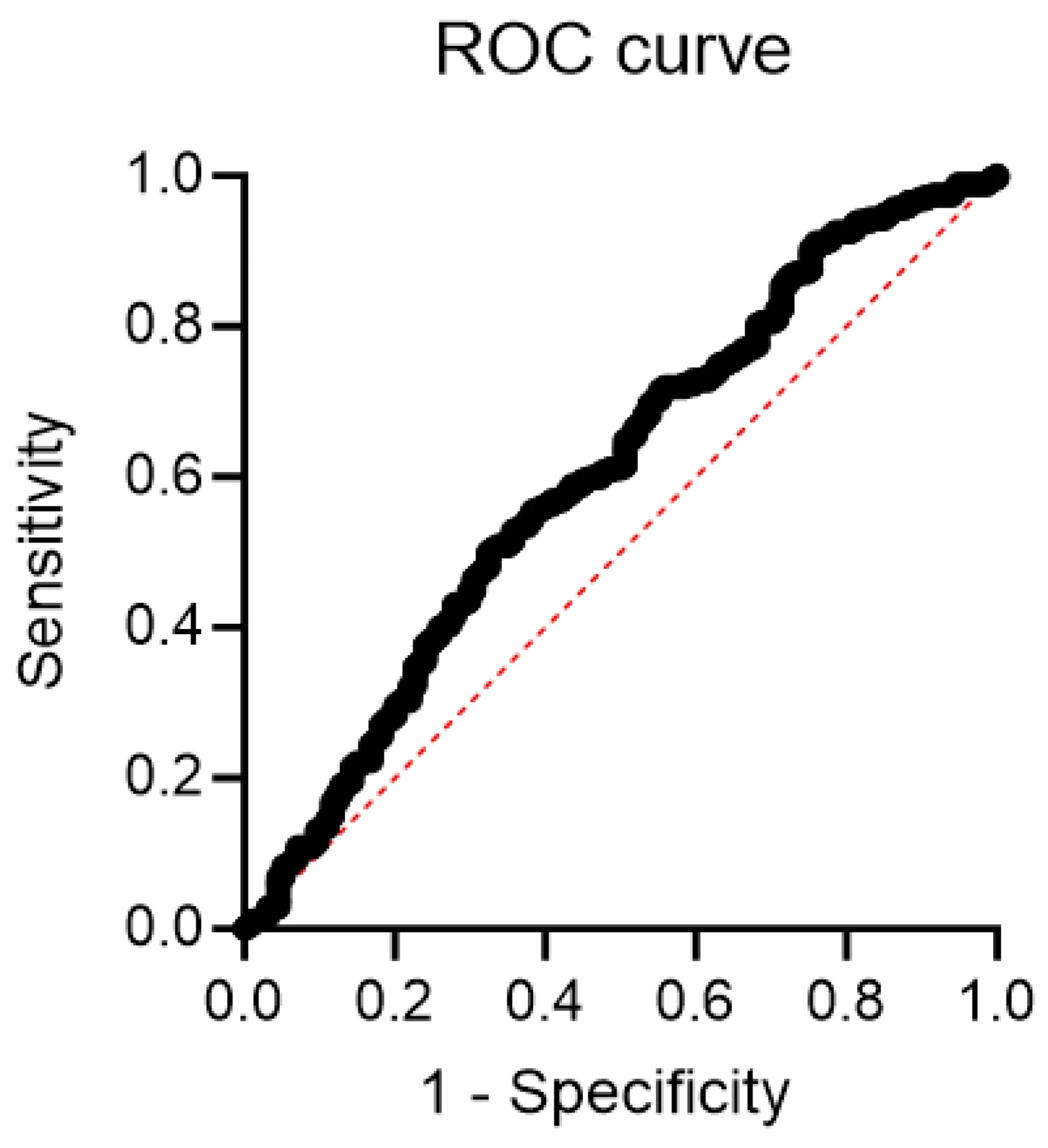
Multivariable logistic regression analysis demonstrated that the PI-RADS score remained independently associated with csPCa after adjusting for age, PSA level, PSAD, and prostate volume (OR = 1.54; 95% CI: 1.12–2.11; *p* = 0.007). The multivariable model demonstrated limited discriminatory performance (AUC = 0.598). ROC, receiver operating characteristic. The black line represents the ROC curve showing the model’s diagnostic performance, while the red dashed diagonal line represents random classification (AUC = 0.5).

Univariable logistic regression showed a significant association between PI-RADS score and the presence of csPCa. Each 1-point increase in PI-RADS score was associated with a 68% increase in the odds of clinically significant prostate cancer (OR = 1.68; 95% CI: 1.26–2.26). In a multivariable analysis including PI-RADS score, age, PSA level, prostate volume, and PSAD, PI-RADS score remained independently associated with csPCa (OR = 1.54; 95% CI: 1.12–2.11). The model demonstrated modest discriminatory performance, with an apparent AUC of 0.598 (95% CI: 0.544–0.653). Bootstrap internal validation yielded a mean optimism of 0.021 and an optimism-corrected AUC of 0.577. Addition of PI-RADS to the clinical model increased the AUC from 0.582 to 0.598 and significantly improved model fit (*p* = 0.007), although the gain in discrimination was small. The ROC curve for the csPCa model is shown in Figure 1 and illustrates model discrimination.

Histopathological upgrading between biopsy and radical prostatectomy specimens was observed in 164 patients (29.1%), whereas concordance between biopsy and postoperative pathology was observed in 305 patients (54.1%). Patients in the upgrade group were slightly older and had significantly higher PSA levels than those in the non-upgrade group. No significant differences were observed in PSAD or prostate volume. Detailed clinicopathological characteristics of patients with and without upgrading are presented in Table 3. The frequency of histopathological upgrading differed significantly according to biopsy Gleason score (*p* < 0.001). Upgrading occurred in 52.0% of patients with biopsy Gleason score 6, compared with 19.5% with Gleason score 7, 15.6% with Gleason score 8, and 13.2% with Gleason score 9.

After adjustment for baseline biopsy Gleason score and the other clinical covariates, PI-RADS remained independently associated with histopathological upgrading (OR = 1.89; 95% CI: 1.31–2.73; *p* < 0.001). The model without PI-RADS had an AUC of 0.722, which increased to 0.750 (95% CI: 0.701–0.795) after inclusion of PI-RADS. Addition of PI-RADS significantly improved model fit (*p* < 0.001), although the improvement in discrimination was modest. Bootstrap internal validation yielded an optimism-corrected AUC of 0.728. Multicollinearity diagnostics showed no evidence of substantial multicollinearity, with VIF values ranging from 1.04 to 2.88. A sensitivity analysis excluding patients with histopathological downgrading yielded consistent results, with PI-RADS remaining independently associated with upgrading. Each 1-point increase in PI-RADS score was associated with an 89% increase in the odds of upgrading on the final histopathological assessment. A sensitivity analysis restricted to patients with PI-RADS 3–5 yielded consistent results for both csPCa and histopathological upgrading. Alternative model specifications using either PSA and prostate volume or PSAD alone yielded materially unchanged estimates for the association of PI-RADS with both study outcomes. The proportion of patients with csPCa increased across PI-RADS categories 3–5, from 42.9% to 67.4% and 75.0%, respectively. In contrast, upgrading rates were 28.6%, 24.1%, and 37.5% across PI-RADS categories 3, 4, and 5, respectively, indicating a non-linear pattern with the highest upgrading rate observed in PI-RADS 5 (Table 4).

## 4. Discussion

This study evaluated the association of preoperative mpMRI and PI-RADS assessment with csPCa and histopathological upgrading after radical prostatectomy. The PI-RADS was significantly associated with both csPCa and the risk of upgrading on postoperative histopathological assessment. In multivariable analyses, PI-RADS remained independently associated with both outcomes. However, given the modest discriminatory performance of the models, these findings should be interpreted as evidence of an independent association rather than strong standalone predictive ability.

The widespread use of mpMRI and standardized PI-RADS assessment has significantly influenced the diagnostic pathway for prostate cancer [13]. Conventional systematic biopsy is constrained by sampling error and may underestimate tumor grade, particularly for anteriorly located lesions or tumors with heterogeneous histological architecture. Several major studies have demonstrated that mpMRI enhances the detection of clinically significant prostate cancer while reducing the identification of clinically insignificant disease. The PROMIS trial showed that mpMRI has significantly higher sensitivity and negative predictive value for csPCa compared with standard TRUS-guided biopsy, suggesting that mpMRI may serve as an effective triage tool before biopsy [3]. Similarly, the PRECISION trial demonstrated superior detection of clinically significant prostate cancer with MRI-targeted biopsy compared with systematic biopsy alone, while also reducing unnecessary biopsies and the overdiagnosis of low-risk disease [4].

Our findings align with those of previous reports showing an increasing probability of csPCa across higher PI-RADS categories. The probability of csPCa increased across PI-RADS categories, with PI-RADS retaining an independent association with csPCa after multivariable adjustment. Importantly, the PI-RADS remained statistically significant after adjusting for PSA, PSAD, prostate volume, and age. This observation supports the concept that MRI provides independent diagnostic information beyond standard clinical parameters [14,15,16]. Although PI-RADS was independently associated with csPCa, the overall discriminatory performance of the predictive models remained limited. The csPCa model showed limited discriminatory performance, whereas inclusion of baseline biopsy grade substantially improved discrimination of the upgrading model. Importantly, addition of PI-RADS provided statistically significant incremental information in both analyses, although the absolute improvement in AUC was modest. These findings support an independent association of PI-RADS with the studied outcomes but do not support its use as a standalone predictive tool. These findings suggest that PI-RADS alone may not be sufficient for precise individual risk prediction. Similar observations have been reported in previous studies evaluating MRI-based risk-stratification models. The limited diagnostic accuracy observed in this study likely reflects the multifactorial nature of prostate cancer biology and inherent limitations in imaging interpretation. Interobserver variability, lesion heterogeneity, prostate size, and the coexistence of inflammatory or benign changes may influence MRI performance.

A substantial proportion of patients showed an increase in histopathological Grade Group between biopsy and radical prostatectomy. Nearly one-third of patients were upgraded between the biopsy and radical prostatectomy specimens, whereas more than half of those with a biopsy Grade Group 1 disease were upgraded on the final pathology. These findings confirm that biopsy-based grading may underestimate disease aggressiveness in a substantial proportion of patients [17,18]. Similar upgrading rates have been reported in previous studies, particularly among patients initially classified as low-risk disease candidates.

Importantly, the PI-RADS was independently associated with the risk of upgrading. Patients in higher PI-RADS categories had increased odds of histopathological underestimation on biopsy. These findings may have clinical implications, particularly for treatment selection and active surveillance. Although patients with PI-RADS 3 lesions are frequently considered to have intermediate-risk imaging findings, this study showed that upgrading remained relatively common even in this subgroup. Therefore, MRI findings should be interpreted cautiously, especially in patients initially diagnosed with Gleason score 6 disease [19,20]. The role of mpMRI in predicting pathological upgrade remains an area of ongoing research. Several authors have suggested that MRI-visible lesions are more likely to harbor aggressive pathological features, including a higher ISUP Grade Group, extracapsular extension, and increased tumor volume. Nonetheless, the predictive performance of MRI for upgrading varies across studies. Differences in study populations, biopsy techniques, MRI protocols, and pathological definitions may have contributed to these discrepancies. Thus, the limited predictive performance observed in this study is consistent with the currently available literature.

After adjustment for other variables, neither PSA nor PSAD showed an independent association with csPCa. Although PSAD differed significantly between the csPCa-positive and csPCa-negative groups in univariable comparisons, it lost statistical significance after MRI findings were included. This observation may suggest that MRI-derived assessments capture clinically relevant tumor characteristics more effectively than conventional serum biomarkers alone. Previous studies have also indicated that mpMRI provides additional diagnostic information beyond PSA-based risk assessment. Nevertheless, PSA and PSAD remain important clinical tools and should be interpreted in conjunction with imaging findings.

The study also benefited from a relatively large cohort in which MRI–TRUS fusion biopsy results could be directly compared with the final pathological findings after radical prostatectomy. Second, using postoperative pathology as the reference standard improved the reliability of the upgrading analysis. Finally, this study reflects real-world clinical practice, which may improve the generalizability of the findings.

This study had some limitations. The retrospective design introduces a potential risk for selection bias. MRI examinations were interpreted by multiple radiologists without systematic independent double reading. Therefore, interobserver agreement could not be assessed. The single-center design may limit the generalizability of these findings to other clinical settings. The multicenter nature of MRI acquisition and the lack of consistently available information regarding MRI scanner strength may have introduced heterogeneity in image acquisition. MRI readers had access to PSA and DRE findings, and pathology reviewers had access to MRI reports and clinical information; therefore, complete blinding could not be ensured. Another limitation was the models’ limited discriminatory performance, suggesting that additional clinical, radiological, or molecular parameters may be needed to improve the prediction of csPCa. The retrospective dataset did not contain sufficiently standardized information on MRI lesion characteristics, including lesion size and location, biopsy core involvement, or clinical stage, precluding their reliable inclusion in the multivariable models. The study cohort included only patients who underwent radical prostatectomy, which may have enriched the population with clinically significant disease and limited the generalizability to biopsy-naive or surveillance populations.

Future studies should focus on integrating mpMRI findings with advanced predictive tools, including radiomics, artificial intelligence models, genomic biomarkers, and nomogram-based approaches. Recent advances in AI-assisted MRI interpretation have demonstrated promising results for improving prostate cancer detection and reducing unnecessary biopsies [21]. Integrating imaging and molecular data may further refine individualized risk stratification in patients with suspected prostate cancer.

Overall, PI-RADS showed significant associations with clinically significant prostate cancer and histopathological upgrading in the radical prostatectomy cohort. mpMRI appears to provide valuable information for preoperative risk assessment and may improve the identification of patients at risk for biopsy underestimation. Despite its limited predictive performance, MRI-based assessments remain an important component of contemporary prostate cancer diagnostics.

## 5. Conclusions

In this retrospective, surgically selected cohort, higher PI-RADS categories were associated with clinically significant prostate cancer and ISUP Grade Group upgrading between biopsy and radical prostatectomy. The multivariable models demonstrated limited discriminatory performance, and the observed associations should not be generalized to biopsy-naive or non-surgical populations. Further studies incorporating baseline biopsy Grade Group and additional biopsy characteristics in broader cohorts are warranted.

## Figures and Tables

**Table 1:** Baseline demographic, clinical, radiological, and pathological characteristics of the study cohort.

Variable (*n* = 564)	Mean ± SD (Median; 25%; 75%)
Age [years]	67.7 ± 7.01 (69.0; 64.0; 73.0)
PSA [ng/mL]	9.1 ± 8.93 (6.91; 5.1; 9.9)
Prostate volume [mL]	45.8 ± 22.5 (41; 32; 54)
Gleason Score Biopsy (6/7/8/9)	(179/302/45/38)
Highest PI-RADS (1/2/3/4/5)	(1/2/42/319/200)
(Upgrading/Downgrading/Concordance)	(164/95/305)
PSAD (ng/mL/cc)	0.233 ± 0.267 (0.167; 0.113; 0.266)
Gleason ≤ 3 + 3 (ISUP 1)	179
Gleason ≥ 3 + 4 (ISUP ≥ 2) = csPCa	385

Continuous variables are reported as mean ± SD and median (25th–75th percentile). Categorical variables are presented as absolute numbers. Available data were age, *n* = 564; PSA, *n* = 552; prostate volume, *n* = 511; PSAD, *n* = 503; biopsy Gleason score, *n* = 564; and PI-RADS, *n* = 564. Missing data were not imputed. PSA, prostate-specific antigen; PSAD, prostate-specific antigen density; PI-RADS, Prostate Imaging Reporting and Data System.

**Table 2:** Demographic, clinical, and radiological characteristics of patients with clinically insignificant prostate cancer (csPCa-negative; ISUP Grade Group 1) versus clinically significant prostate cancer (csPCa-positive; ISUP Grade Group ≥ 2).

Variable (*n* = 564)	csPCa Negative (*n* = 179)	csPCa Positive (*n* = 385)
Age [years]	68.2 ± 6.38 (69.0; 64.3; 73.0)	67.5 ± 7.28 (68.0; 64.0; 73.0) ns
PSA [ng/mL]	8.45 ± 8.03 (6.63; 5; 9.28)	9.39 ± 9.3 (7; 5.1; 10) ns
Prostate volume [mL]	48.4 ± 22.2 (42.5; 34; 56.3)	44.7 ± 22.5 (39.4; 31; 53) ns
PSAD (ng/mL/cc)	0.204 ± 0.24 (0.147; 0.104; 0.217)	0.246 ± 0.277 (0.176; 0.117; 0.283) **
Surgery Duration [minutes]	179 ± 54.8 (174; 145; 206)	157 ± 47.4 (149; 125; 180) ***
Margin [mm]	4.22 ± 11.6 (1.5; 0.5; 3)	4.66 ± 4.96 (2.5; 1; 7) ns
PI-RADS (1/2/3/4/5)	(0/1/24/104/50)	(1/1/18/215/150) ns

Continuous variables are reported as mean ± SD, with median and interquartile range (IQR: 25th–75th percentile). Categorical variables are reported as counts. Statistical significance was assessed using the unpaired Student *t* test or Mann–Whitney U test, as appropriate. PSA, prostate-specific antigen; PSAD, prostate-specific antigen density; PI-RADS, Prostate Imaging Reporting and Data System. *** *p* < 0.001, ** *p* < 0.01, ns—not significant.

**Table 3:** Comparison of demographic and clinicopathological characteristics between patients with histopathological upgrading and non-upgrading following radical prostatectomy.

Variable (*n* = 564)	Non-Upgrade (*n* = 400)	Upgrade (*n* = 164)
Age [years]	67.03 ± 7.03 (68.0; 63.3; 72.0)	68.7 ± 6.89 (70; 65; 73) *
PSA [ng/mL]	8.57 ± 7.3 (6.68; 5; 9.3)	10.4 ± 11.9 (7.7; 5.4; 12) *
PSAD (ng/mL/cc)	0.233 ± 0.27 (0.164; 0.114; 0.260)	0.245 ± 0.26 (0.175; 0.116; 0.286)
Prostate volume [mL]	44.6 ± 21.6 (40; 32; 52)	49 ± 24.2 (42; 32; 60)

Upgrading was defined as an increase of at least one ISUP Grade Group from biopsy to final radical prostatectomy pathology. The non-upgrade group included concordant and downgraded cases. Continuous variables are presented as mean ± SD and as median with interquartile range (IQR: 25th–75th percentile). Statistical significance was assessed using an unpaired Student *t* test or the Mann–Whitney U test, as appropriate. PSA, prostate-specific antigen; PSAD, prostate-specific antigen density. * *p* < 0.05.

**Table 4:** Distribution of csPCa and histopathological upgrading by PI-RADS category.

PI-RADS	csPCa	Upgrade
1 (*n* = 1)	1 (100%)	0 (0%)
2 (*n* = 2)	1 (50%)	0 (0%)
3 (*n* = 42)	18 (42.9%)	12 (28.6%)
4 (*n* = 319)	215 (67.4%)	77 (24.1%)
5 (*n* = 200)	150 (75%)	75 (37.5%)

Clinically significant prostate cancer was defined as ISUP Grade Group ≥ 2 (Gleason score ≥ 3 + 4). Upgrading was defined as an increase of at least one ISUP Grade Group from biopsy to final radical prostatectomy pathology. Values are reported as *n* (%) within each PI-RADS category. csPCa, clinically significant prostate cancer; PI-RADS, Prostate Imaging Reporting and Data System.

## Data Availability

The data used in this study are available upon reasonable request from the authors.

## Abbreviations

The following abbreviations are used in this manuscript:

AUC	Area under the curve
CI	Confidence interval
csPCa	Clinically significant prostate cancer
DRE	Digital rectal examination
IQR	Interquartile range
ISUP	International Society of Urological Pathology
MRI	Magnetic resonance imaging
mpMRI	Multiparametric magnetic resonance imaging
OR	Odds ratio
PI-RADS	Prostate Imaging Reporting and Data System
PSA	Prostate-specific antigen
PSAD	Prostate-specific antigen density
ROC	Receiver operating characteristic
SD	Standard deviation
TRUS	Transrectal ultrasound
